# Validity of the CORE Wearable Sensor During Internal Cooling Induced by Hyperhydration with Cold Water at Rest

**DOI:** 10.3390/s26165042

**Published:** 2026-08-08

**Authors:** Eric D. B. Goulet, Antoine Jolicoeur Desroches, Thomas A. Deshayes, Timothée Pancrate, Marc Elouann Pidoux, Antoine Carmichael, Tristan Etienne

**Affiliations:** 1 Faculty of Physical Activity Sciences, University of Sherbrooke, Sherbrooke, QC J1K 2R1, Canada; antoine.jolicoeur-desroches@usherbrooke.ca (A.J.D.); thomas.deshayes@usherbrooke.ca (T.A.D.); timothee.pancrate@usherbrooke.ca (T.P.); marc.elouann.pidoux@usherbrooke.ca (M.E.P.); antoine.carmichael@usherbrooke.ca (A.C.); tristan.etienne@usherbrooke.ca (T.E.); 2 Performance, Hydration and Thermoregulation (PHT) Laboratory, University of Sherbrooke, Sherbrooke, QC J1K 2R1, Canada; 3 Research Centre on Aging, University of Sherbrooke, Sherbrooke, QC J1H 4C4, Canada

**Keywords:** cold water ingestion, exercise, heat flux sensors, heat stress, pre-exercise cooling, wearable sensors

## Abstract

Pre-exercise internal cooling through cold-water-induced hyperhydration may attenuate increases in core body temperature (T_C_) and improve endurance performance. Quantifying the extent of reduction in T_C_ induced by hyperhydration is important for optimizing its timing before exercise. We compared T_C_ values obtained from a wearable sensor, the CORE, with those of a gastrointestinal temperature telemetric sensor (G_TS_) during hyperhydration. Eleven participants (two women; age: 24 ± 4 yrs) completed a 120 min seated period where they consumed, over the first 60 min, four boluses of 4 °C water (7.5 mL · kg fat-free mass [FFM]^−1^), each containing 0.35 g · kg FFM^−1^ of glycerol. Measures of T_C_ were taken every 20 min with both sensors. According to the G_TS_, hyperhydration induced a peak T_C_ decline of −0.76 ± 0.31 °C at min 60; at this time, the change in T_C_ from baseline estimated by the CORE was −0.09 °C ± 0.20 °C. The greatest decline in T_C_ detected by the CORE was −0.11 ± 0.19 °C. Bland and Altman analyses revealed that average T_C_ declines of –0.1, −0.2, −0.3, −0.4, −0.5 and −0.6 °C from baseline were associated with T_C_ values estimated by the CORE that were respectively +0.29, +0.41, +0.57, +0.65, +0.77 and +0.89 °C higher than the G_TS_. An intraclass correlation coefficient of 0.12 suggested poor agreement between instruments. These results raise concerns about the ability of the CORE to detect changes in T_C_ induced by a hyperhydration protocol generating a substantial heat sink.

## 1. Introduction

Exercise performed in hot and humid conditions, particularly when interspersed with periods of high-intensity efforts, can predispose athletes to marked elevations in core body temperature (T_C_), which could potentially compromise endurance performance [1]. Various strategies can be used by athletes to attenuate the increases in T_C_ and better sustain performance under such scenarios; pre-exercise internal body cooling via the ingestion of large volumes of cold fluid (hyperhydration) or ice slurry is one of them [2]. Indeed, hyperhydration and ice slurry ingestion have been shown to lower T_C_ before exercise [3,4,5,6]. Moreover, the volume of cold substances entering the body creates a heat sink that enables increasing body heat storage capacity. In turn, this can potentially allow greater physical work to be done before reaching a T_C_ that compromises the maintenance of an exercise intensity judged satisfactory to sustain performance [2].

Monitoring the impact of a pre-exercise internal body cooling strategy on the reduction in T_C_ is important and may have important implications from a performance perspective [7,8]. First, it can help athletes choose the most effective pre-exercise internal body cooling strategy possible. Next, understanding the kinetics of the reduction in T_C_ associated with a given internal body cooling strategy may help optimize its ingestion timing so that exercise begins in a state of maximal cooling. Finally, it may help athletes plan an optimal pacing strategy for the upcoming exercise. Indeed, the magnitude of the reduction in T_C_ induced by an internal body cooling strategy could be used by athletes as an indicator to estimate how long they may be able to sustain an exercise intensity that they would not normally dare to maintain under conditions in which the body had not been cooled before exercise.

Because of either their invasive nature, need for trained personnel, lack of practicality, the discomfort they may cause, or their high cost, traditionally used and accepted methods to monitor changes in T_C_ during clinical and exercise settings [9,10,11], i.e., rectal, gastrointestinal or esophageal sensors, cannot be used routinely and on a large scale in field settings. Nevertheless, wearable sensors are now available to provide estimates of T_C_ under real world context [12,13], and these devices could be used by athletes to monitor the impact of pre-exercise internal body cooling. The CORE (greenteg, Zürich, Switzerland) is a commercially available, compact (40 mm width × 50 mm height × 8.35 mm depth), lightweight (12 g), rechargeable, and relatively low-cost wearable sensor designed to estimate T_C_ non-invasively. The device is worn on the skin ~20 cm below the axilla and has attracted a great deal of scientific interest over the past 5 yrs. Indeed, it has been the object of several validity and reliability studies conducted under various settings such as during intensive care, sleeping, free living, resting and exercise conditions [14,15,16,17,18,19,20,21,22,23,24,25,26,27,28,29].

To the best of our knowledge, no study has yet examined the validity of the CORE sensor under resting conditions involving cold-water-induced hyperhydration, which produces a substantial internal heat sink. This seems relevant, as athletes represent part of the target market of the manufacturer of the CORE sensor, thereby rendering them susceptible to use the device under conditions of internal body cooling. The CORE derives estimates of T_C_ based on an algorithm relying on heart rate, the single-heat-flux method and skin temperature [12,25]. Internal body cooling may reduce T_C_ while producing limited changes in skin heat flux, skin temperature and heart rate [5,30]. Consequently, the CORE sensor may have difficulty detecting changes in T_C_ under these specific conditions.

Interestingly, our laboratory has recently demonstrated that the CORE was insensitive to a 0.26 °C decline in T_C_ induced by the ingestion of 7.5 mL · kg fat-free mass (FFM)^−1^ of cold water (4 °C) [19]. However, since the amount of water ingested was small and the decline in T_C_ remained within the superior range of the normal daily variation in gastrointestinal or rectal temperatures, i.e., ±0.25 °C [31], and the mean bias between instruments was lower than the ±0.27 °C value often regarded as worthwhile in the literature [9,10], it would be premature and inadequate to infer from these results regarding the ability of the CORE to detect decreases in T_C_ induced by potent internal body cooling interventions.

Wearable sensors designed to estimate T_C_ are increasingly used because they provide a practical and non-invasive alternative to conventional methods. As these devices become more widely adopted, it is essential not only to validate their accuracy under conditions for which they were developed, but also to identify the physiological conditions under which their performance may deteriorate. Defining these operational boundaries is critical for ensuring appropriate interpretation of sensor-derived measurements and for guiding future improvements in wearable sensing technology. Therefore, the purpose of this study was to compare the changes in T_C_ measured by a gastrointestinal temperature telemetric sensor (G_TS_) and a wearable sensor developed to estimate T_C,_ the CORE, during a hyperhydration period at rest designed to induce a substantial decline in T_C_. It was hypothesized that T_C_ measured by the CORE and the G_TS_ would differ by more than the established threshold for a worthwhile practical difference between instruments, i.e., ±0.27 °C.

## 2. Materials and Methods

### 2.1. Participants

Eleven individuals (2 women) participated in this study. Table 1 presents the volunteers’ individual demographic, anthropometric, and body composition characteristics. Volunteers’ identification numbers correspond to those presented in Figure 3 to assist in the interpretation of the individual responses. Collectively, their mean age, height, body mass, body mass index, body surface area, fat mass and FFM were respectively 24 ± 4 yrs, 175 ± 9 cm, 68 ± 10 kg, 22.3 ± 2.4 kg · m^−2^, 1.8 ± 0.2 m^2^, 9.3 ± 4.5 kg and 59 ± 10 kg. Prior to participating in the study volunteers were required to read a consent form and subsequently a verbal and written consent was obtained. The study was approved by the CIUSSS Estrie-CHUS Ethics Committee (2020-3606). The women completed the experiments in the follicular phase of their menstrual cycle.

### 2.2. Procedures

#### Context of the Study

The current findings represent a secondary analysis of results from a study that compared the effect of pre-exercise hyperhydration and euhydration during a subsequent 5 km running time trial performance [19]. Herein, we report results pertaining only to the hyperhydration phase and describe the technical procedures only relevant to the current study.

### 2.3. Preliminary Visit

Volunteers completed a preliminary visit where height (standard stadiometer), body mass (BX-300+, Atron Systems, West Caldwell, NJ, USA), fat mass and FFM (Lunar Prodigy, GE Healthcare, Chicago, IL, USA) were measured. At the end of the visit volunteers were required to return to the laboratory at 7-day intervals and always at the same time of day to complete two experimental trials administered in a randomized, counterbalanced and crossover fashion: one in which they began a 5 km running time trial in a euhydrated state, and another in which they began the running time trial in a hyperhydrated state.

### 2.4. Experiments

Prior to each experiment, diet, training, sleeping and hydration were controlled for, as previously explained [32]. Immediately after arriving at the laboratory, volunteers voided their bladder, collected a urine sample, body mass was measured, a heart rate chest strap was attached to the torso and the temperature and humidity level of the laboratory were measured (Extech H30, Flir, Goleta, CA, USA). Then, participants were seated for a 10 min stabilization period, after which measures of heart rate and T_C_ derived from the G_TS_ and CORE were taken. Following these procedures, volunteers began a 120 min seated period during which they consumed, every 20 min over the first 60 min, four boluses of 4 °C water, each provided at a dose of 7.5 mL · kg FFM^−1^ with 0.35 g · kg FFM^−1^ of glycerol [33]. Every 20 min thereafter, measures of heart rate, T_C_, urine volume and body mass (post-void) were taken.

### 2.5. Measurements

Core body temperature was measured with G_TS_ (CorTemp, HQ Inc., Palmetto, FL, USA). Gastrointestinal temperature telemetric sensors can provide continuous, real-time, minimally invasive T_C_ monitoring, with applications in healthcare, sports, wellness and occupational safety [34]. When compared to rectal temperature, the G_TS_ has been demonstrated to represent a valid index of T_C_ [35]. To avoid any interaction with fluid intake, volunteers ingested the G_TS_ 10 h prior to their arrival at the laboratory [36,37]. Gant, Atkinson [37] reported no apparent differences between rectal and gastrointestinal temperatures after the repeated ingestion of 4 °C water before and within bouts of exercise when the G_TS_ were ingested 10 h prior to experiment commencement. Similarly, when the G_TS_ were ingested 8 h before the onset of experiment, O’Brien, Goosey-Tolfrey [38] demonstrated that the consumption of 6.8 g · kg^−1^ of ice slurry during recovery following passive heating did not produce any relevant differences between the fall in rectal and gastrointestinal temperatures. Gastrointestinal temperature telemetric sensor signals were recorded with a single CorTemp Data Recorder (HQ Inc, Palmetto, FL, USA). According to the manufacturer’s specifications, the sensor has a sensitivity range of 0–50 °C, a stated accuracy of ±0.1 °C, and a resolution of 0.01 °C [39,40]. Each G_TS_ was calibrated before the beginning of the experiment. Calibration was performed at four different temperatures (37, 38, 39, and 40 °C) in a heated bath (Precision 281, Thermo Scientific, Waltham, MA, USA) using a high-precision, partial-immersion, non-mercury glass thermometer (Thermco Ertco, Fisher Scientific, Pittsburgh, PA, USA). A 4-point regression line was used to predict the gastrointestinal temperature telemetric sensor temperature [41]. A single CORE wearable sensor, bought in April 2021, was used for the current study. As recommended by the manufacturer, the CORE was (1) clipped to the chest strap, ~20 cm below the axilla, (2) was applied on the skin 10 min before the first baseline measurements, in accordance with the manufacturer’s recommendation at the time the study was conducted and; (3) operated paired with signals from an external Garmin heart rate monitor throughout all experimental trials. Consequently, heart rate data were available to the CORE sensor during T_C_ estimation. Over the course of data collection for the current study, 3 firmware updates were performed on the CORE, i.e., (1) version 0.3.17 to 0.4.0, (2) version 0.4.0 to 0.4.1, and (3) version 0.4.1 to 0.6.0. Despite the firmware updates, the algorithm used to estimate T_C_ always remained the same throughout the study (personal communication with the manufacturer). Moreover, another personal communication with a representant of the manufacturer of the CORE on 23 June 2026 indicates that the changes made to the algorithm since the current study ended would not change the capacity of the CORE to estimate changes in T_C_ produced by internal body cooling strategies. The CORE wearable sensor estimates T_C_ non-invasively using a proprietary algorithm that integrates information derived from heat flux, skin temperature, and heart rate. Although the implementation of the commercial algorithm has not been publicly disclosed, a previous study has demonstrated that a predictive model combining these physiological measurements can accurately estimate T_C_ [42]. The heat flow sensing technology underlying the device is described in the manufacturer’s patent [43]. According to the manufacturer’s specifications, the CORE estimates T_C_ over a measurement range of 36–42 °C with a mean absolute deviation of 0.21 °C and 95% limits of agreement of ±0.55 °C. Apparently, the device has primarily been developed for continuous, non-invasive estimation of T_C_ during field and laboratory applications, including exercise, occupational heat stress monitoring, and physiological research [44]. Heart rate was measured using a Garmin Premium chest electrode (Garmin, Olathe, KS, USA). Urine-specific gravity was assessed using a digital refractometer (PAL-10S, Atago, Bellevue, WA, USA). Urine volume was measured with a graduate urinal. Body mass was evaluated with a high-precision digital platform scale (BX-300+, Atron Systems, West Caldwell, NJ, USA). Body surface area was computed using the Dubois and Dubois equation [45]. Accumulated fluid retention at each measurement timepoint was computed by subtracting the accumulated urine volume from the accumulated fluid intake.

### 2.6. Statistical Analysis

A linear mixed-effects model with random intercepts for volunteers was used to analyze the effect of time and sensors as well as their interaction on T_C_. Time, sensors and their interaction were treated as fixed effects. A linear mixed-effects model with random intercepts for volunteers was used to analyze the effect of time on accumulated fluid retention, urine volume, and heart rate. Time was treated as a fixed effect. For the above analysis, we used a scaled identity covariance structure to model the repeated measurements. Moreover, a variance components matrix was selected for the covariance structure of the random intercepts. The distribution of residuals was verified with a Shapiro–Wilk test as well as visualization of the Q-Q plot. If relevant, post hoc analyses were performed using the false discovery rate procedure [46]. Data for these analyses are presented as mean ± standard deviation (SD). Statistical significance was considered as *p* ≤ 0.05. For describing the relative validity of the CORE, a repeated measures correlation [47], as well as an intraclass correlation coefficient (two-way mixed, absolute agreement, single measure) were computed. The correlation coefficient was interpreted as specified by Akoglu [48] and the intraclass correlation coefficient as specified by Koo and Li [49]. For description of absolute validity, the bias in measurement between sensors, the typical within-subject variation in measurement and Bland and Altman 95% limits of agreement, were calculated. The bias between sensors represents the difference between two comparisons. The typical within-subject variation in measurement was taken as the square root of the residual covariance obtained from the linear mixed-effects model comparing sensors [50]. For the Bland and Altman-related data, we first examined the relationship over time between the differences in T_C_ observed between sensors and their averages. A marked heteroscedastic pattern was identified, indicating that the bias between sensors was dependent upon the changes in T_C_. Therefore, as recommended by Bland and Altman, a regression approach for non-uniform differences was applied to determine the biases and associated standard deviations [51]. Because repeated measurements were performed within individuals over time with both sensors, a linear mixed-effects model with random intercepts for volunteers was used to derive the regression equations and associated standard deviations between the sensor’s differences and their averages, as specified by Myles and Cui [52]. The average T_C_ between sensors was taken as the covariable. A variance components matrix was selected for the covariance structure of the random intercepts. No additional covariance structure was specified for the repeated measurements, thereby allowing estimation of a single residual variance for the differences around the predicted bias. Two Bland and Altman plots were generated. The first examined the relationship between the average T_C_ values of the G_TS_ and the CORE and the differences in T_C_ between the CORE and the G_TS_ over the entire 120 min sitting period. The maximal decline in T_C_ measured by the G_TS_ occurred at min 60. Hence, agreement between the CORE sensor and the G_TS_ was also examined for changes between min 60 and baseline. For each timepoint (i.e., 20, 40 and 60 min), changes in T_C_ were calculated relative to baseline for each device, and Bland and Altman analyses were performed on the differences between changes. This approach allowed us to determine whether the CORE sensor accurately tracked the magnitude of the reduction in T_C_ induced by cold-water-induced hyperhydration. A T_C_ difference of ±0.27 °C or 95% limits of agreement of ±0.27 °C between sensors was considered practically meaningful [9,10,53,54]. Analyses were conducted using Microsoft Excel (version 2502, Redmond, WA, USA), R studio (version 2026.01.2, Posit Team) and IBM SPSS Statistics (version 21, New York, NY, USA).

## 3. Results

### 3.1. Ambient Conditions, Hydration State at Arrival at the Laboratory, Glycerol Ingestion, Fluid Intake, Urine Production, Accumulated Fluid Retention, and Heart Rate

Mean temperature and relative humidity during the sitting period were 21.8 ± 0.3 °C and 58.0 ± 6.7%, respectively. Volunteers were apparently well hydrated at their arrival at the laboratory. Indeed, the mean urine-specific gravity value (1.013 ± 0.007) suggested that the kidneys were not in a state of significant water conservation. Volunteers ingested a total of 82.3 ± 13.7 g of glycerol (1.2 ± 0.1 g · kg body mass^−1^) with 1763 ± 293 mL (25.9 ± 2.0 mL · kg body mass^−1^) of cold water. There was a significant time effect for urine production, accumulated fluid retention, and heart rate (all *p* < 0.01). Urine production peaked at min 60 with a volume of 292 ± 116 mL, which was significantly different from times 0, 20, 40, 100 and 120 (all *p* < 0.01).

**Figure 1:**
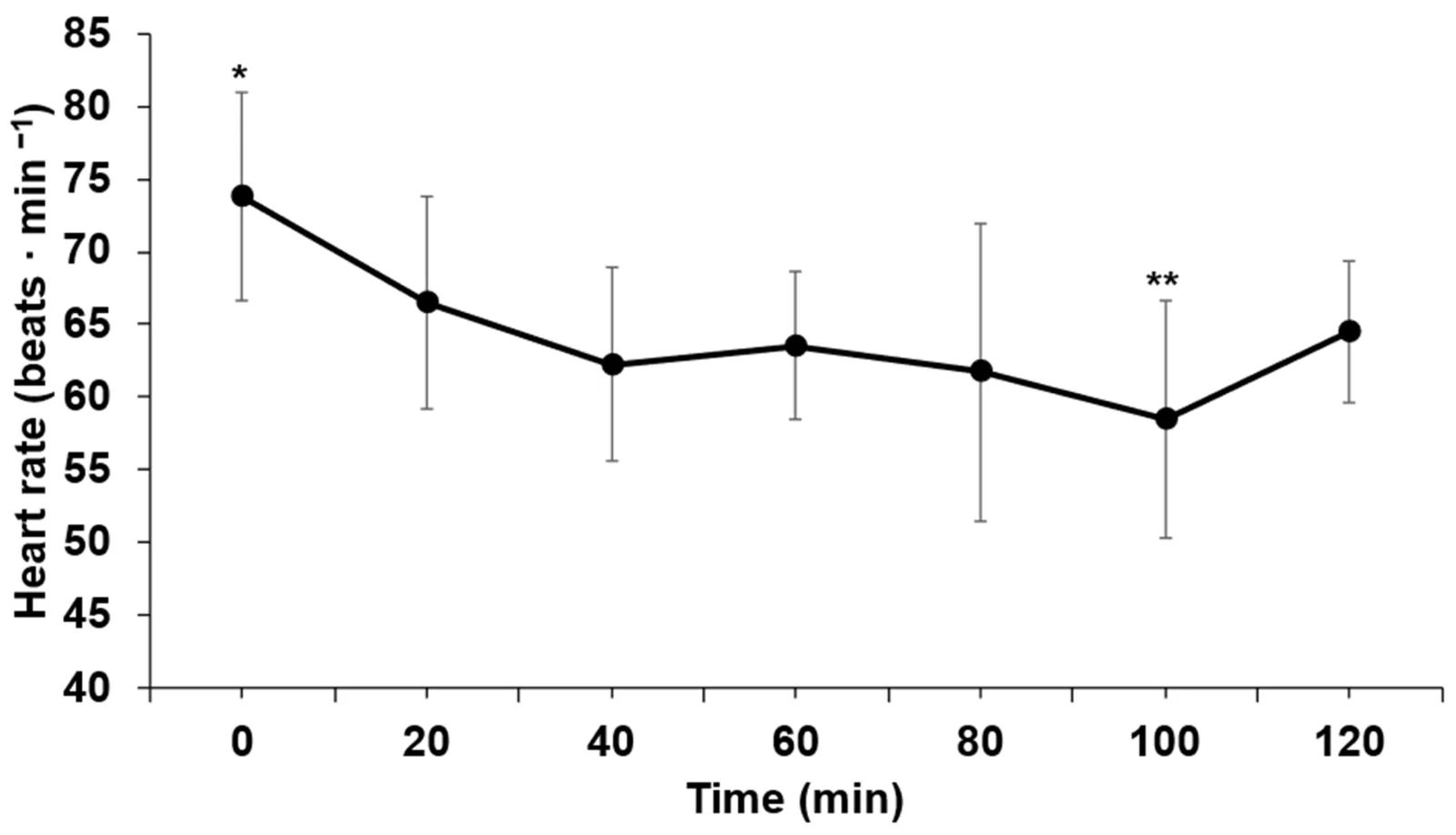
Changes in heart rate during the 120 min sitting period. * = significantly different from all other timepoints, *p* < 0.05. ** = significantly different from min 20 and 120, *p* < 0.05. Results are mean ± SD.

Accumulated fluid retention increased continuously until min 80 where it culminated at 1014 ± 541 mL. This volume was significantly greater than those observed at 0, 20, 40, 100 and 120 min (all *p* ≤ 0.03). After 120 min, accumulated fluid retention reached 645 ± 546 mL, with a total volume of urine produced of 1143 ± 418 mL. Heart rate changes during the sitting period are shown in Figure 1. Heart rate decreased from 74 ± 7 to 62 ± 7 beats · min^−1^ during the first 40 min of the sitting period after which it remained relatively stable fluctuating between 58 and 64 beats · min^−1^. Post hoc comparisons revealed a significant difference between min 0 and all other timepoints (all *p* < 0.01) and min 100 with timepoints 20 and 120 min (both *p* < 0.02).

### 3.2. Comparison Between the Gastrointestinal Temperature Telemetric Sensor and CORE Data

**Figure 2:**
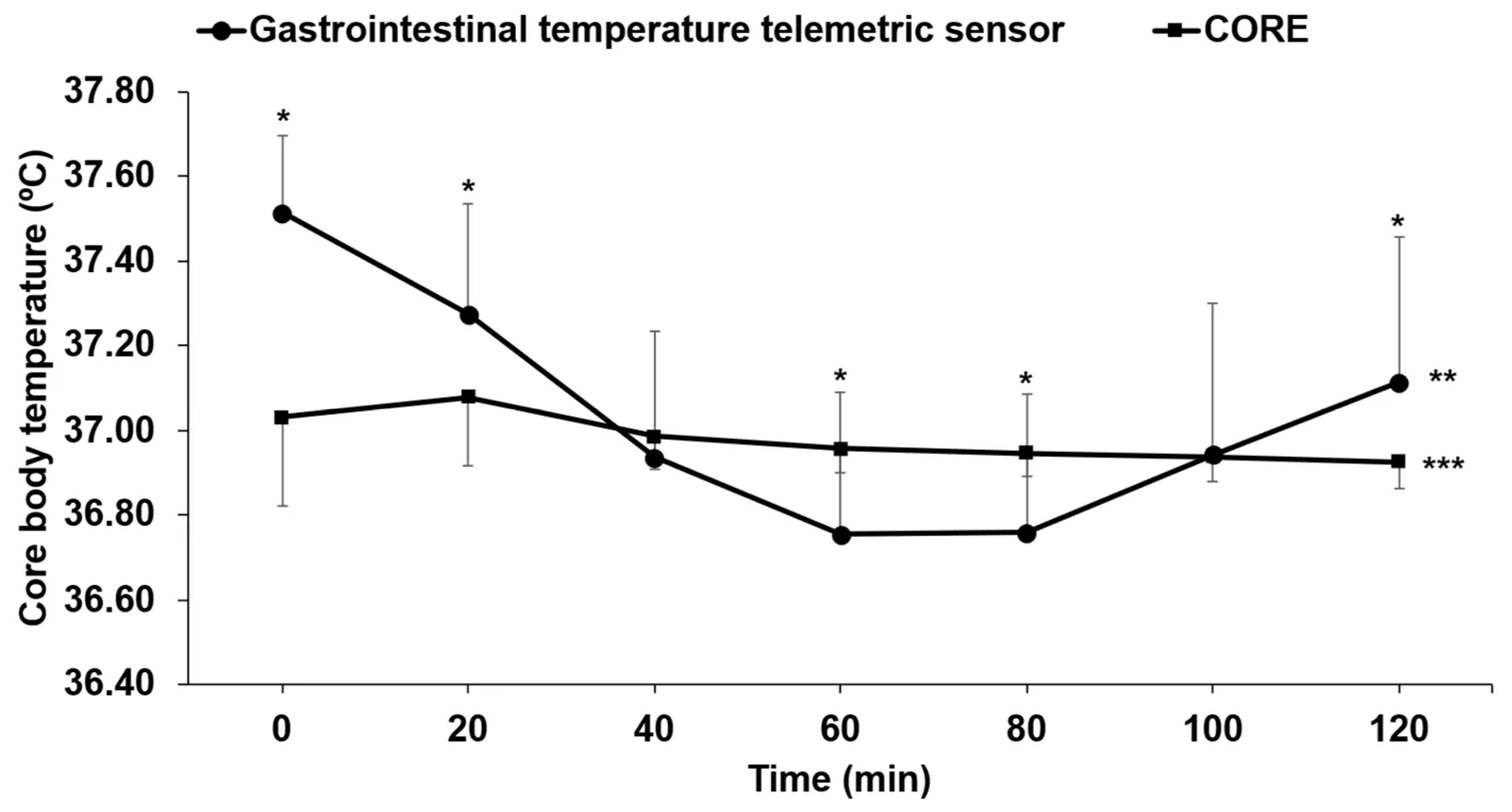
Changes in core body temperature monitored with the gastrointestinal temperature telemetric sensor and the CORE during the 120 min sitting period. * = *p* < 0.05 compared with the CORE at the same timepoint. ** = all timepoints significant different from min 0, *p* < 0.05. *** = no timepoint significantly different from min 0, *p* > 0.05. Results are mean ± SD.

Figure 2 shows the changes in T_C_ monitored with the G_TS_ and CORE during the 120 min sitting period. Statistically significant effects of time (*p* < 0.01), sensors (*p* = 0.046) and interaction (*p* < 0.01) were observed.

Hyperhydration was successful in achieving internal body cooling. In fact, the G_TS_ demonstrated a change in T_C_ of −0.76 ± 0.31 °C between min 0 and 60 (*p* < 0.01). On the other hand, the change in T_C_ from min 0 to 60 with the CORE was only of −0.09 °C ± 0.20 °C (*p* = 0.35). The peak decline in T_C_ captured by the CORE was of −0.11 ± 0.19 °C, which occurred at min 120. The CORE demonstrated no significant change in estimated T_C_ throughout the hyperhydration period, compared with baseline (all *p* ≥ 0.19). On the other hand, with the G_TS_, all timepoints differed significantly from the baseline value (all *p* < 0.01). Mean T_C_ during the entire 120 min sitting period was 37.04 ± 0.39 °C with the G_TS_, and 36.98 ± 0.12 °C with the CORE (*p* = 0.046). Post hoc analyses revealed a significant difference between sensors at min 0, 20, 60, 80 and 120 (all *p* ≤ 0.02).

**Figure 3:**
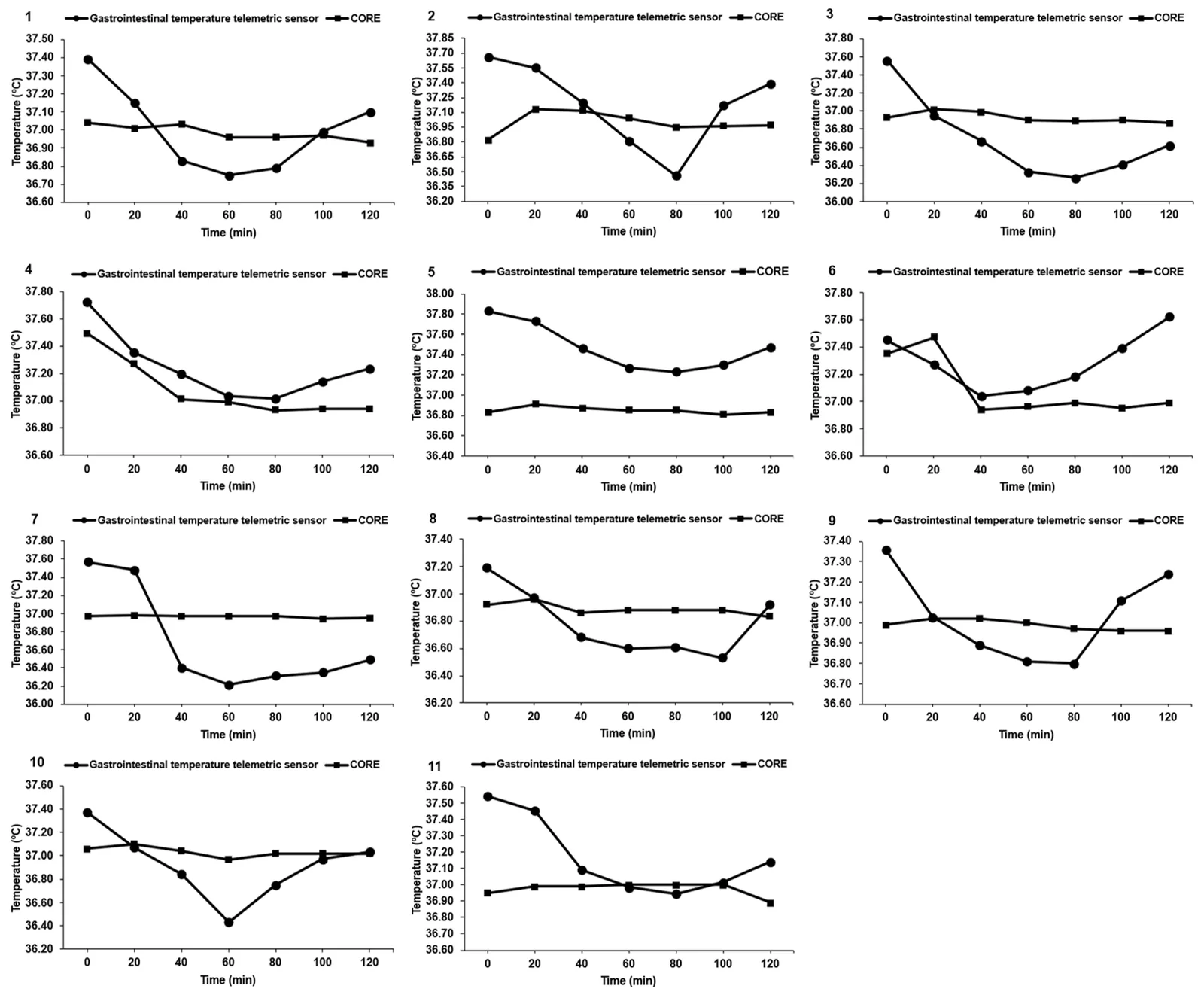
Individual responses of all 11 volunteers to the hyperhydration periods, as measured by the gastrointestinal temperature telemetric sensors and the CORE. Panels 6 and 7 represent data for the two women.

For all comparisons combined, i.e., 0–120 min, the typical within-subject variation in measurement was of the order of 0.19 °C, the repeated measures correlation between sensors was not statistically significant (*p* = 0.07) and poor with a *r_rm_* = 0.22, and the intraclass correlation coefficient was also poor at 0.12 (*p* = 0.15). Figure 3 shows the individual responses of all 11 volunteers to the hyperhydration periods, as measured by the G_TS_ and the CORE. Only two volunteers, 4 and 6, showed a response with the CORE that appeared consistent with the initial pattern of change in T_C_ expected following the use of an internal body cooling strategy, namely a relatively sharp decline in T_C_. In the remaining nine volunteers, fluctuations in CORE-derived T_C_ relative to baseline were minimal throughout the hyperhydration period. Panels 6 and 7 represent the data for women.

**Figure 4:**
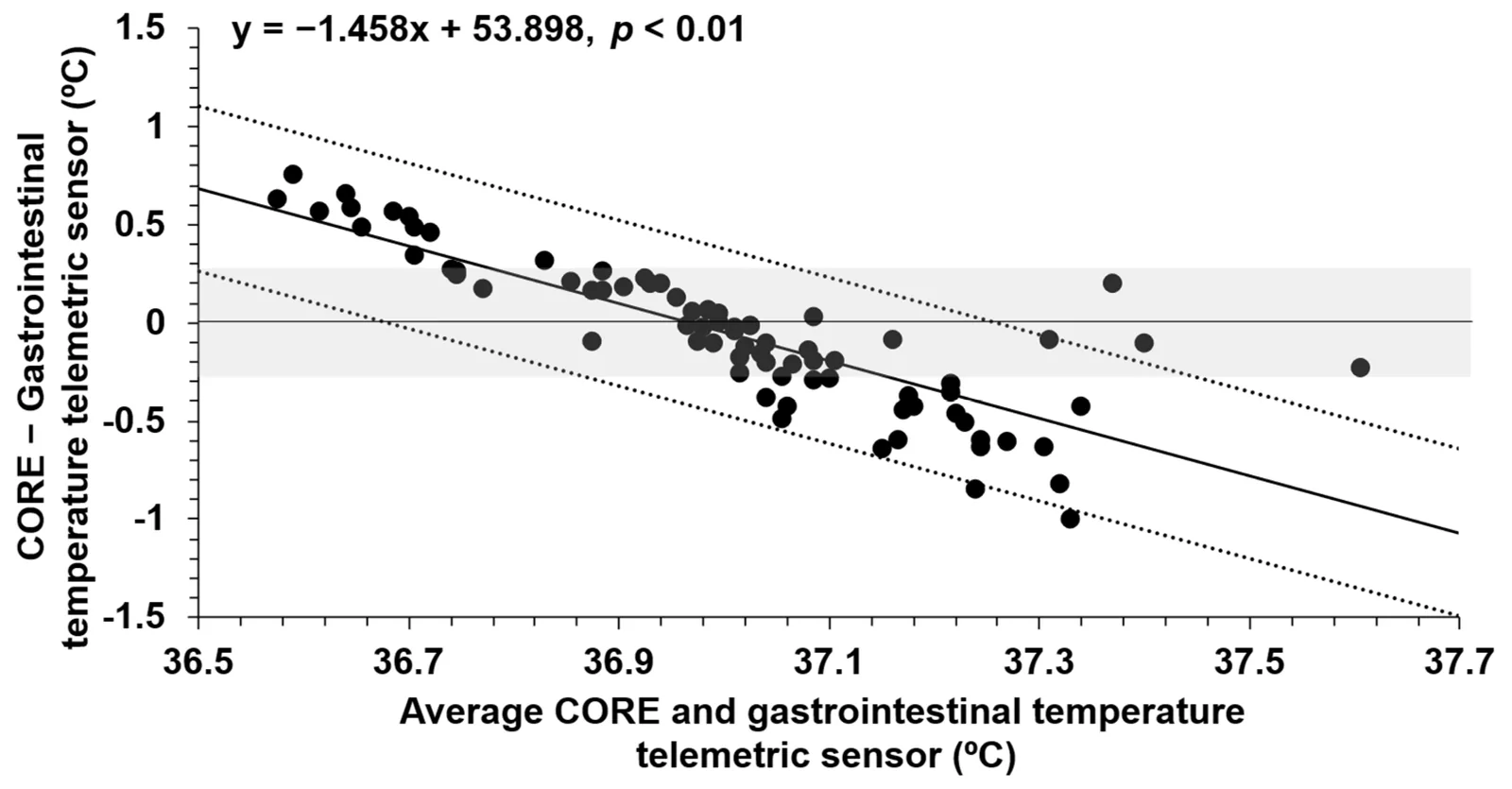
Bland and Altman plot showing the relationship between the average core body temperature measured with the CORE and gastrointestinal temperature telemetric sensor and the magnitude of the differences in core body temperature observed between the CORE and the gastrointestinal temperature telemetric sensor during the entire 120 sitting period. The black horizontal line represents the line of zero difference between sensors. The grey area represents the zone of acceptable difference (±0.27 °C) in core body temperature between sensors. The dotted lines represent ±1.96 × SD around the regression line.

Figure 4 illustrates a Bland and Altman plot showing the relationship between the averages T_C_ measured with the G_TS_ and CORE and the magnitude of the differences in T_C_ observed between the CORE and the G_TS_.

These results indicate that the bias between the CORE and the G_TS_ was inversely proportional to the average value of T_C_ measured by the CORE and the G_TS_. More specifically, based on the line of no difference between sensors, the CORE started overestimating T_C_ below an average T_C_ of ~36.97 °C, whereas the opposite was observed at T_C_ above 36.97 °C. Nevertheless, between average T_C_ values of ~36.78 and 37.14 °C, the CORE showed no measurement difference compared with the G_TS_ that fell within the range considered unacceptable from a physiological standpoint, i.e., ±0.27 °C. In the context of Figure 4, 55% of the differences between the G_TS_ and the CORE were within ±0.27 °C.

**Figure 5:**
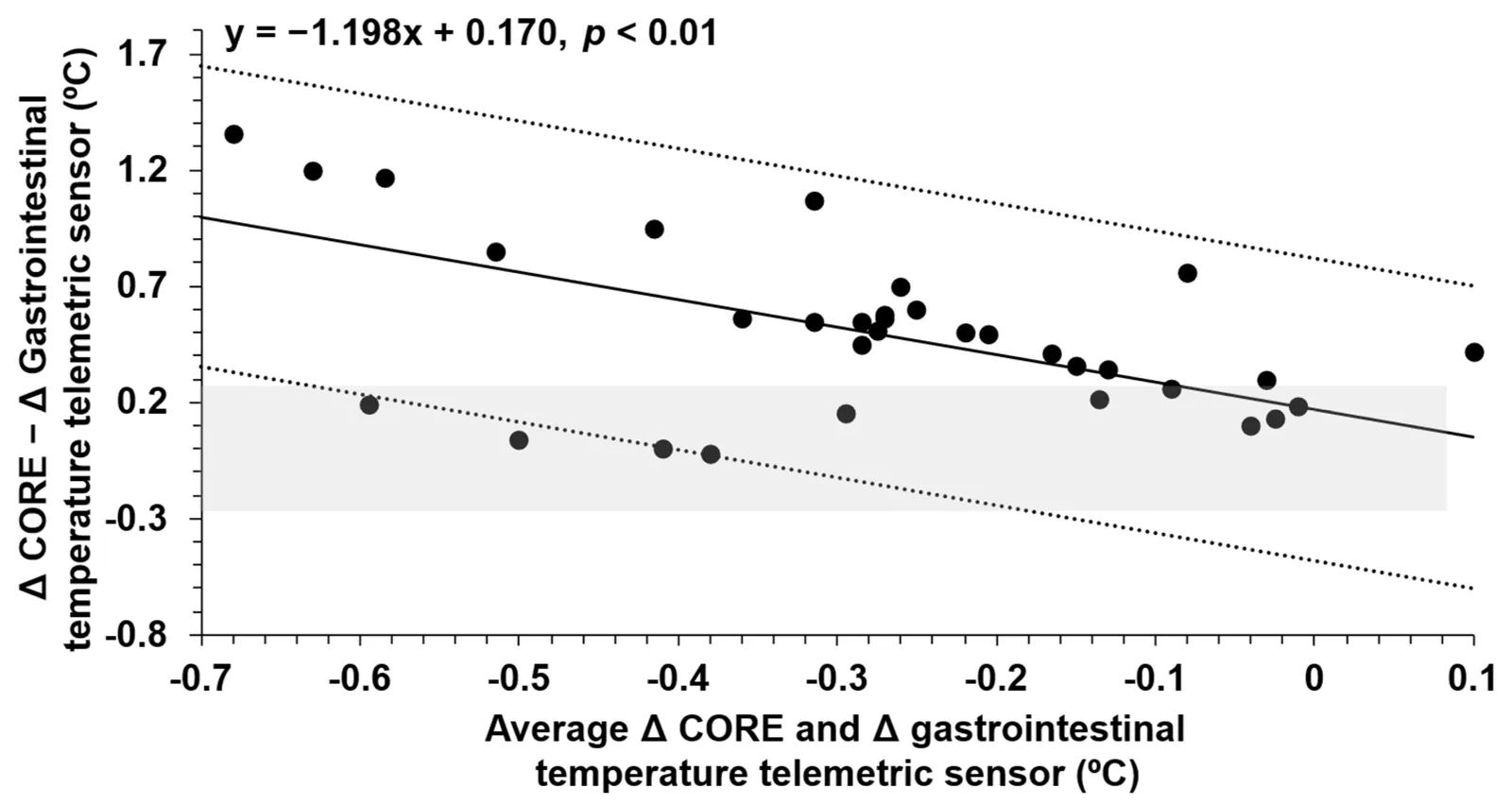
Bland and Altman plot showing agreement between the CORE sensor and the gastrointestinal temperature telemetric sensor for changes in core body temperature from baseline during the first 60 min of the hyperhydration period. At each 20 min interval, the mean change from baseline between the two sensors is plotted against the difference in change from baseline between sensors (ΔTCORE − ΔG_TS_). The grey area represents the zone of acceptable difference (±0.27 °C) in core body temperature between sensors. The dotted lines represent ±1.96 × SD around the regression line.

The Bland and Altman plot shown in Figure 5 indicates that the CORE had difficulty capturing the substantial declines in T_C_ that occurred during the first 60 min of the seated period. Indeed, for average T_C_ declines of −0.1, −0.2, −0.3, −0.4, −0.5 and −0.6 °C from baseline, the T_C_ values estimated by the CORE were respectively +0.29 (95% limits of agreements, −0.36–0.94 °C), +0.41 (−0.24–1.06 °C), +0.57 (−0.08–1.22 °C), +0.65 (0.00–1.30 °C), +0.77 (0.12–1.42 °C), and +0.89 °C (0.24–1.54 °C) higher than the G_TS_. Finally, during the first 60 min of the sitting period, only 30% of the T_C_ differences between the ΔG_TS_ and the ΔCORE fell within limits considered irrelevant from a practical perspective.

## 4. Discussion

This study aimed to determine whether the CORE wearable sensor could detect the decline in T_C_ induced by a potent internal body cooling strategy, specifically cold-water-induced hyperhydration. The main finding was that, despite a marked reduction in G_TS_-derived T_C_ during the first 60 min of the sitting period, the CORE showed difficulties in detecting the declines in T_C_. More specifically, while the G_TS_ detected a substantial decline in T_C_ of 0.76 °C from min 0 to 60, the corresponding change measured by the CORE was only of −0.09 °C. These results raise doubts about the ability of the CORE to detect rapid reductions in T_C_ induced by hyperhydration with cold water at rest.

The present findings extend previous observations from our laboratory, which suggested that the CORE showed limited ability to detect a modest 0.26 °C reduction in T_C_ induced by the ingestion of a smaller dose of cold water [19], i.e., 7.5 mL · kg FFM^−1^ at 4 °C. In that earlier context, any conclusion that the CORE was likely unable to detect this decrease in T_C_ remained largely speculative and could not reasonably be extended to potent internal body cooling strategies, given that the magnitude of the observed change was at the lower limit of the normal daily variation in T_C_ (i.e., ±0.25 °C, [31]) and lower than the ±0.27 °C value often regarded as worthwhile in the literature [9,10,31].

In the present study, however, a peak hyperhydration level of 1014 mL of 4 °C water combined with glycerol induced a much larger decline in T_C_, i.e., −0.76 °C, thereby providing a stronger test of the device’s ability to detect internal body cooling. Despite this pronounced decline in T_C_, the CORE still showed problems to reflect the magnitude and kinetics of the reduction in T_C_ demonstrated by the G_TS_. To provide support to this observation, the mixed-effects linear model revealed a clear statistically significant effect for interaction (i.e., *p* < 0.01) between sensors despite the small sample size of the current study. The repeated measures correlation assessing the association between the T_C_ of the two sensors was poor, thereby suggesting that the responses of the sensors did not move strongly together. The intraclass correlation coefficient was also poor, indicating that agreement between sensors was not strong. Finally, among the eleven individual responses reported, only two suggest that the CORE may detect declines in T_C_ following hyperhydration. Taken together, these findings suggest that the limitations of the CORE are potentially not only restricted to detecting very small changes in T_C_ induced by internal body cooling strategies, but also extend to larger declines that are clearly meaningful from both a physiological and practical perspective. For the interested readers, Table 2 provides a descriptive comparison of the G_TS_ and CORE performances during cold-water-induced hyperhydration between the present study and that of Jolicoeur Desroches, Naulleau [19]. These comparisons can be used to highlight the consistently limited response of the CORE sensor across two distinct magnitudes of gastrointestinal temperature reduction induced by cold water hyperhydration, which likely suggests the limited viability of the CORE sensor under scenarios of internal body cooling. Indeed, the present hyperhydration protocol induced a decline in T_C_, as measured with the G_TS_, that exceeds or is in line with data reported in other studies evaluating the impact of cold-water-induced hyperhydration or ice slurry ingestion on T_C_ at rest [55,56].

One important observation from the current study is that the bias between the CORE and the G_TS_ was not constant across the range of the average T_C_ measured. This heteroscedastic pattern is particularly problematic because it indicates that the disagreement between sensors is not random, but rather systematically related to the underlying T_C_. For instance, close examination of the Bland and Altman illustrated in Figure 4 reveals that the CORE began to overestimate T_C_ below a mean T_C_ of ~36.97 °C and to underestimate it above this threshold. Moreover, data deriving from the Bland and Altman depicted in Figure 5 indicate that average T_C_ declines of between −0.1 to −0.6 °C from baseline over the first 60 min of hyperhydration were associated with T_C_ values estimated by the CORE that were all above changes suggesting a decline in T_C_, as well as being above the acceptable agreement between sensors of ±0.27 °C. These observations are contrary to what should be expected of temperature sensors from an accuracy standpoint following the use of a potent internal body cooling strategy, i.e., be able to detect a decrease in T_C_. From an applied standpoint, these results suggest that the CORE could provide the false impression that T_C_ is stable during cold-water-induced hyperhydration when it is in fact decreasing meaningfully. This limitation may be especially relevant in the context where athletes or practitioners seek to monitor the effectiveness of a pre-exercise internal body cooling strategy and optimize its timing before competition.

The lack of sensitivity of the CORE to internal body cooling likely relates, at least in part, to the nature of the physiological signals on which its algorithm is based. Indeed, the device estimates T_C_ from heart rate, the single-heat-flux technique, and skin temperature. The role of heart rate in the algorithm used by the CORE to estimate the changes in T_C_ during exercise is likely substantial. Indeed, during exercise, increases in metabolic heat production, which dictate the magnitude of T_C_ elevation [57], are closely associated with rises in heart rate [58]. Accordingly, algorithms have previously been developed to estimate T_C_ during exercise using changes in heart rate [59,60]. In the present study, however, substantial changes in T_C_ occurred concomitantly with only relatively trivial changes in heart rate, as shown in Figure 1. Thus, the contribution of heart rate to the prediction of T_C_ changes could only be limited, as supported by our findings. The effect of cold fluid or ice slurry ingestion on mean skin temperature under resting conditions in thermoneutral conditions appears to be relatively trivial [4,5,61]. Therefore, this observation suggests that this variable could not have played an important role in the estimation of the changes in T_C_ by the CORE in the current study, although we recognize that we did not monitor skin temperature during the hyperhydration period. Finally, considering our results, it must be acknowledged that skin heat flux likely played a negligible role in the ability of the CORE to estimate the changes in T_C_ in the context of the present study. This is not surprising, as internal body cooling acts to extract heat internally and, as a result, heat flux from the core to the skin decreases. Accordingly, our results suggest that the CORE may have difficulties dealing with decreases in skin heat flux, at least with those of the magnitude produced by hyperhydration with cold water. Altogether, these results suggest that the current algorithm used by the CORE may not be fully suited to predicting changes in T_C_ occurring during internal cooling at rest.

Another noteworthy finding of the current study is that only 30% of T_C_ differences between sensors from baseline values during the first 60 min of cooling were within ±0.27 °C. This observation reinforces the conclusion that the CORE lacked acceptable agreement with the G_TS_, and this precisely during the period when accurate monitoring was most needed. In addition, and importantly, although the difference in average T_C_ over the 120 min sitting period was trivial between sensors, this lack of difference should not be construed as evidence of validity, as the CORE showed difficulties in adequately reproducing the true temporal changes in T_C_ measured by the G_TS_.

This study has some limitations that must be acknowledged. The sample size was relatively small and included only 11 participants. Therefore, all findings of the current study must be interpreted with this in mind. For instance, results found in Figure 3 suggest that two individuals exhibited declines in CORE-estimated T_C_ that closely paralleled those observed with the G_TS_, which is discrepant to the responses observed in the other individuals. Nevertheless, on a group level, the results reported in Figure 3 suggest a tendency supporting the possibility that the CORE may have difficulty capturing declines in T_C_ during a period of internal body cooling. Additional studies incorporating specific physiological measurements are needed to better characterize the sources of inter-individual variability in the CORE responses under scenarios of internal body cooling. The results of the current study apply specifically to an internal body cooling strategy using hyperhydration at rest in temperate laboratory conditions. It remains to be determined whether the CORE would behave differently with other internal body cooling strategies used under similar or different surrounding temperatures. Oesophageal, rectal, and gastrointestinal temperatures represent different physiological compartments and therefore exhibit different response kinetics [62]. Accordingly, it must be acknowledged that the magnitude of the agreement between the CORE sensor and the reference method may depend on the measurement site selected. Nevertheless, in the present study, the CORE sensor exhibited only minimal changes in estimated T_C_ throughout the internal body cooling intervention, whereas the G_TS_ detected a substantial decrease over the first 60 min. Therefore, although the absolute magnitude of the response may have differed had esophageal or rectal temperature been used as the reference method, it is unlikely that the principal conclusion of the present study would have been altered. Moreover, it must be re-emphasized that gastrointestinal temperature represents a valid index of T_C_ compared with rectal temperature, and that both body areas may respond similarly to internal body cooling [37,38,63]. Although it is currently recommended by the manufacturer of the CORE to allow ~15 min for stabilization after application of the sensor, the recommendation at the time of data collection for this study was 10 min, which was followed in the present study. While a longer stabilization period could have resulted in a slightly different estimated baseline T_C_ by the CORE, the change in CORE-estimated T_C_ during the first hour of hyperhydration was on average −0.09 °C. Consequently, it is unlikely that a slightly longer stabilization period would have materially affected the interpretation of the present findings. The underrepresentation of women (two of 11 participants) is another limitation of the present study, and it precludes any meaningful assessment of sex-related differences in the CORE performance under the current experimental condition. Nevertheless, the individual responses shown in Figure 3 suggest that the two women exhibited changes in T_C_ similar to those of the men.

## 5. Conclusions

In conclusion, the present study suggests that the CORE wearable sensor may have a limited ability to detect the decline in T_C_ induced by pre-exercise hyperhydration with cold water. Therefore, caution may be warranted when using the CORE to assess the effectiveness of cold-water-induced hyperhydration or to inform performance-related decisions following the use of this internal body cooling strategy. Future studies with larger sample sizes are needed to confirm our findings. Moreover, it would be interesting to test the capability of the CORE under scenarios of external body cooling. Rather than questioning the overall utility of the CORE sensor under the scenario of the current study, the present findings should contribute to define the physiological conditions under which this sensing approach should be used with caution. Such information is important for both end users and developers seeking to improve future generations of wearable sensors attempting to estimate T_C_.

## Figures and Tables

**Table 1:** Individual demographic, anthropometric, and body composition characteristics of the volunteers.

Volunteers	Characteristics							
	Gender	Age (yrs)	Height (cm)	Body Mass ** (kg)	Body Mass Index (kg · m^−2^)	Body Surface Area (m^2^)	Fat Mass (kg)	Fat-Free Mass * (kg)
1	Man	21	179	69.2	21.8	1.9	4.5	66.1
2	Man	23	194	85.1	22.5	2.2	8.3	77.6
3	Man	21	173	78.4	26.2	1.9	11.0	68.1
4	Man	30	166	65.5	23.9	1.7	13.7	50.4
5	Man	28	182	70.8	21.4	1.9	9.0	60.0
6	Woman	22	169	53.9	19.0	1.6	7.8	45.7
7	Woman	30	159	58.4	23.1	1.6	13.1	45.9
8	Man	23	176	62.5	20.1	1.8	3.1	59.4
9	Man	20	172	64.2	21.8	1.8	9.1	55.0
10	Man	27	176	59.7	19.2	1.7	4.4	54.9
11	Man	21	178	81.3	25.8	2.0	18.2	63.3

* Fat-free mass excludes bone mineral content. ** Body mass was measured using a high-precision digital platform scale; therefore, fat mass and fat-free mass may not necessarily sum to body mass.

**Table 2:** Descriptive comparison of the gastrointestinal temperature telemetric sensor and CORE performances during cold-water-induced hyperhydration between the present study and reference [19].

Variables	Present Study	Reference [19]
Sample size	11	12
Duration of observation (min)	120	120
Absolute volume of cold water (4 °C) ingested (mL)	1763 ± 293	420 ± 66
Time to peak decrease in gastrointestinal temperature (min)	60	40
Largest detected decrease in core body temperature by the gastrointestinal temperature telemetric sensor at time to peak decrease in gastrointestinal temperature (°C)	−0.76 ± 0.31	–0.26 ± 0.22
Largest detected change in core body temperature by the CORE at time to peak decrease in gastrointestinal temperature (°C)	−0.09 ± 0.20	+0.02 ± 0.10

## Data Availability

The raw data supporting the conclusions of this article will be made available by the authors on request.

## Abbreviations

The following abbreviations are used in this manuscript:

T_C_	Core body temperature
FFM	Fat-free mass
G_TS_	Gastrointestinal temperature telemetric sensor
SD	Standard deviation
